# Using Immersive Technologies to Develop Medical Education Materials

**DOI:** 10.7759/cureus.12647

**Published:** 2021-01-12

**Authors:** Sinem S Ovunc, Musa B Yolcu, Senol Emre, Mehmet Elicevik, Sinan Celayir

**Affiliations:** 1 Department of Pediatric Surgery, Istanbul University-Cerrahpasa, Cerrahpasa Medical Faculty, Istanbul, TUR

**Keywords:** immersive technologies, augmented reality, simulation, medical education, surgical training, three-dimensional (3d) printing, 360-degree video recording, self-paced learning

## Abstract

Principles of modern surgical education for clerkship and residency were established by the novel approaches of Sir William Osler, MD, Flexner report, and Halsted's principles. The evaluation of surgical education has continued to benefit from the wisdom of the past by harnessing technologies. Rapidly changing and improving the nature of the surgery fostered that evaluation and enforced the institutions to find new solutions for surgical education.

In the present descriptive technical report, our aim was threefold: (1) to share acquired educational materials based on immersive technologies involving 3D-printing, Augmented Reality (AR), and 360-degree video recording to improve ongoing pediatric surgery student training at our faculty, (2) to describe workflow underlying the construction of the materials, and (3) to provide approaches that may help other students and lecturers to develop their educational materials.

The educational materials, including 3D-printed models, AR hybrid student book, a hydrogel-based simulation model of the kidney, and Mirror World Simulation, were constructed. The authors, who are medical students, led the construction of the educational materials, so the educational materials were shaped by a collaboration between students and pediatric surgeons.

The materials constructed enabled the students to practice surgical procedures and experience different surgical environments. We believe these educational materials can serve as a valuable resource for training in many medical specialties in the future.

This work was presented at the American College of Surgeons (ACS) Quality and Safety Conference Virtual, August 21-24, 2020.

## Introduction

Medical education has evolved dramatically from ancient times to the present. One of the milestones in that evaluation is the detailed illustrations drawn during the Renaissance, which enabled physicians to explore the human anatomy, make discoveries and inventions in various medical fields, and translate their knowledge to trainees [1-2]. Today, many tools and technologies involving simulations, smartphones, tablets, telemedicine, Augmented Reality (AR), Virtual Reality, 360-degree video recording, wearable devices, digital games, e-learning environments, atlases accompanied by AR, virtual patients, and 3 Dimensional (3D)-printed models are in use to facilitate students' essential knowledge acquisition and help them to gain required skills [3-4]. A 2011 study in *Transactions of the American Clinical and Climatological Association *demonstrated the exponential growth of medical knowledge: in 1950, the doubling time was 50 years; in 1980, 7 years; in 2010, 3.5 years, and is projected to be 73 days by 2020 [5]. In addition to expanding the volume of medical knowledge, the characteristics of trainers have changed over the years through the changes in habits and attitudes. According to Prensky's famous article published in 2001, today's medical students, as he designated as "digital natives," do not just have different hobbies or music preferences; they learn differently as well. Consequently, digital natives have obliged digital immigrants to transform their educational methods and materials [6]. Much more educational materials are in progress to meet the requirements of medical education that have been changing under the influence of many factors involving the changes in pedagogical methods, health care environment, roles of the physician, students' profile, and rapidly increasing volume of medical knowledge [3-6]. In this descriptive technical report, we wanted to describe educational materials developed using immersive technologies to improve ongoing pediatric surgery student training in our faculty [7-9]. We harvested computer-aided design software (CAD), 3D printers, AR development tools, hydrogel-combined molding techniques, and 360-degree video recording to construct our educational materials. Students and surgeons worked collaboratively on the construction of educational material; consequently, they embedded it into the theoretical and practical curriculum of pediatric surgery clerkship.

The purpose of the present paper is not limited to demonstrating educational materials based on immersive technologies. In this paper, we also tried to share the workflow underlying the construction of the materials in a detailed and step-by-step manner as we aim to enable readers worldwide to construct these educational materials at their institutions. Lastly, these instructions were enhanced with videos, and the recordings from clerkship were given to demonstrate the usage of educational materials in daily practice.

## Technical report

1. Construction of 3D-printed models of congenital anomalies

**Figure 1:**
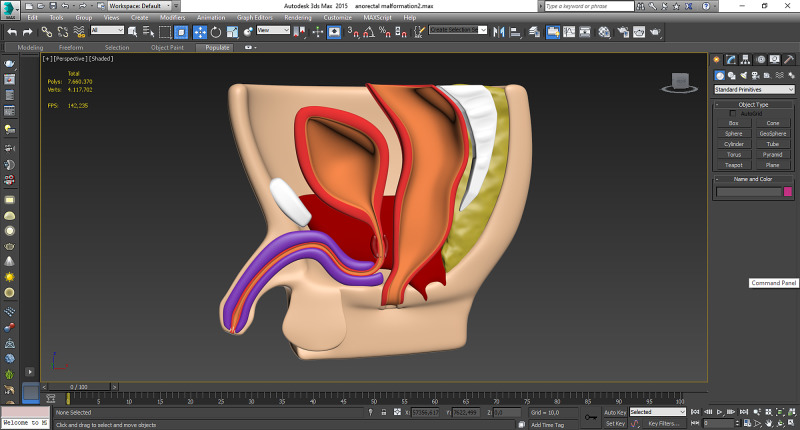
Three-dimensional modeling Anorectal malformation, esophageal atresia, vesicoureteral reflux, choledochal cyst, and jejunoileal atresia were modeled three-dimensionally. The virtual model of the anorectal malformation was demonstrated in this figure.

*(a) Three-Dimensional Modeling:* Five different pathologies in pediatric surgery involving anorectal malformation, esophageal atresia, vesicoureteral reflux, choledochal cyst, and jejunoileal atresia were modeled three-dimensionally and appropriately to anatomical characteristics according to two-dimensional drawings, radiological views, and surgical experience using Autodesk 3ds Max (Autodesk, San Rafael, USA) (Figure 1). Then, 3D models were exported as stereolithography (STL) files. They were imported into Meshmixer (Autodesk, San Rafael, USA) and repaired using that software to prepare them for printing.

**Figure 2:**
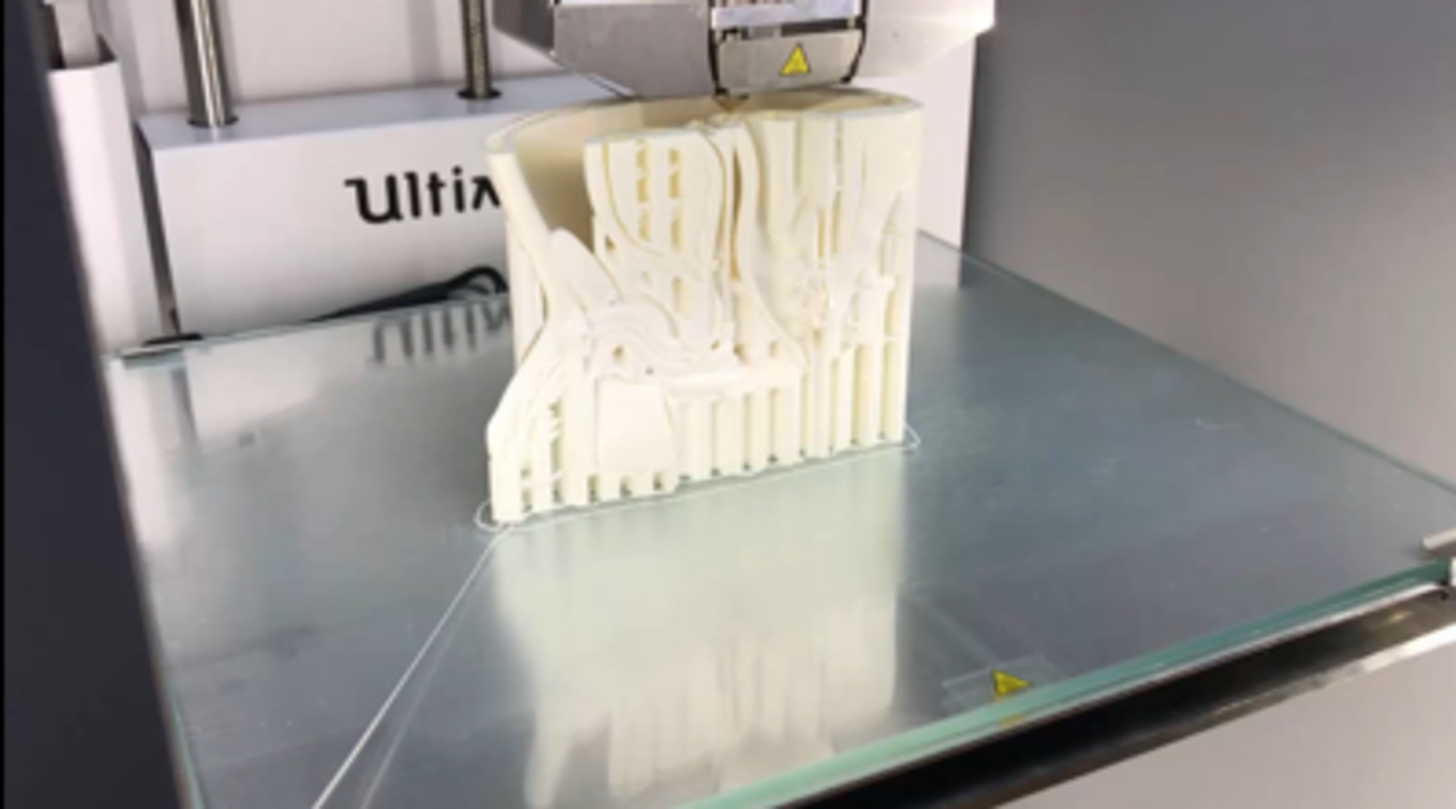
3D printing Models of anorectal malformation, esophageal atresia, vesicoureteral reflux, choledochal cyst, and jejunoileal atresia were 3D printed.

*(b) 3D Printing:* Ultimaker 2 Extended + (Ultimaker B.V., Geldermalsen, The Netherlands) was preferred for printing 3D models. Following the repair process, 3D models were imported into the Ultimaker Cura (Ultimaker B.V., Geldermalsen, The Netherlands), a specific software used to prepare models for printing. The sizes of the models were rearranged according to the characteristics of models to make them more feasible and effective for clinical and educational indications. Then, settings involving printing material (filament type), nozzle speed, and infill/support futures were modified specific to models and saved as G-code files. Subsequently, models were 3D printed on Ultimaker 2 Extended + (Figure 2).

**Figure 3:**
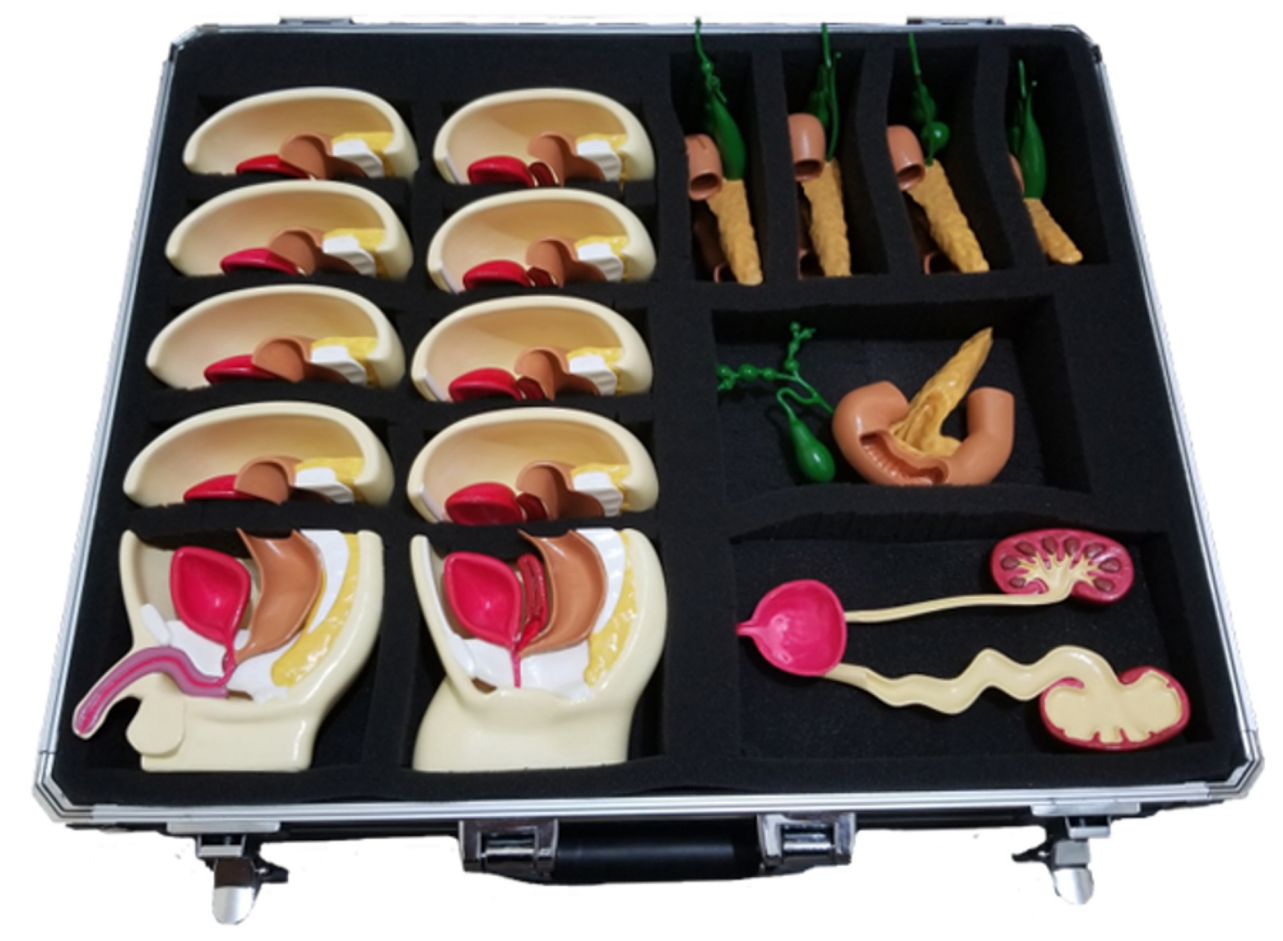
Fine-tuning Models of anorectal malformation, esophageal atresia, vesicoureteral reflux, choledochal cyst, and jejunoileal atresia were colored manually.

*(c) Fine-tuning:* As mentioned above, the models were supported with materials added by Ultimaker Cura to make the printing easier. After the printing step had been completed, the models were cleared from support materials. Then they were colored to differentiate different parts. That enabled us to demonstrate and teach pathologies more effectively (Figure 3).

2. Construction of the simulation model based on 3D printing and hydrogel 

**Figure 4:**
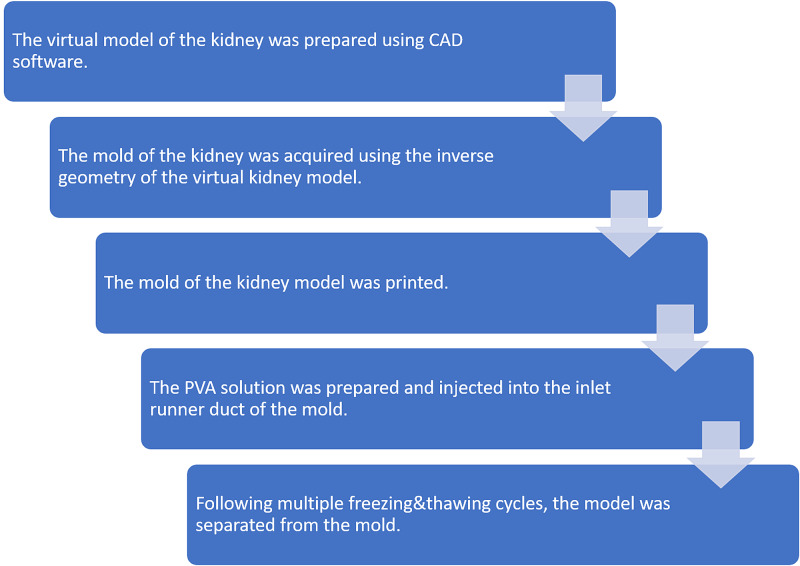
The construction of the simulation model based on 3D printing and hydrogel was summarized using a process flow diagram. CAD: computer-aided design; PVA: polyvinyl alcohol

Figure 4 shows the process of constructing the simulation model based on 3D printing and hydrogel. 

**Figure 5:**
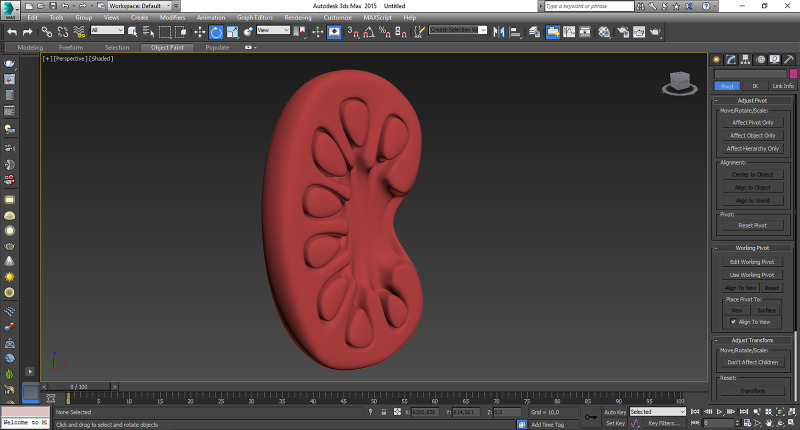
Designing the kidney model for the external mold Virtual model of the kidney was prepared.

**Figure 6:**
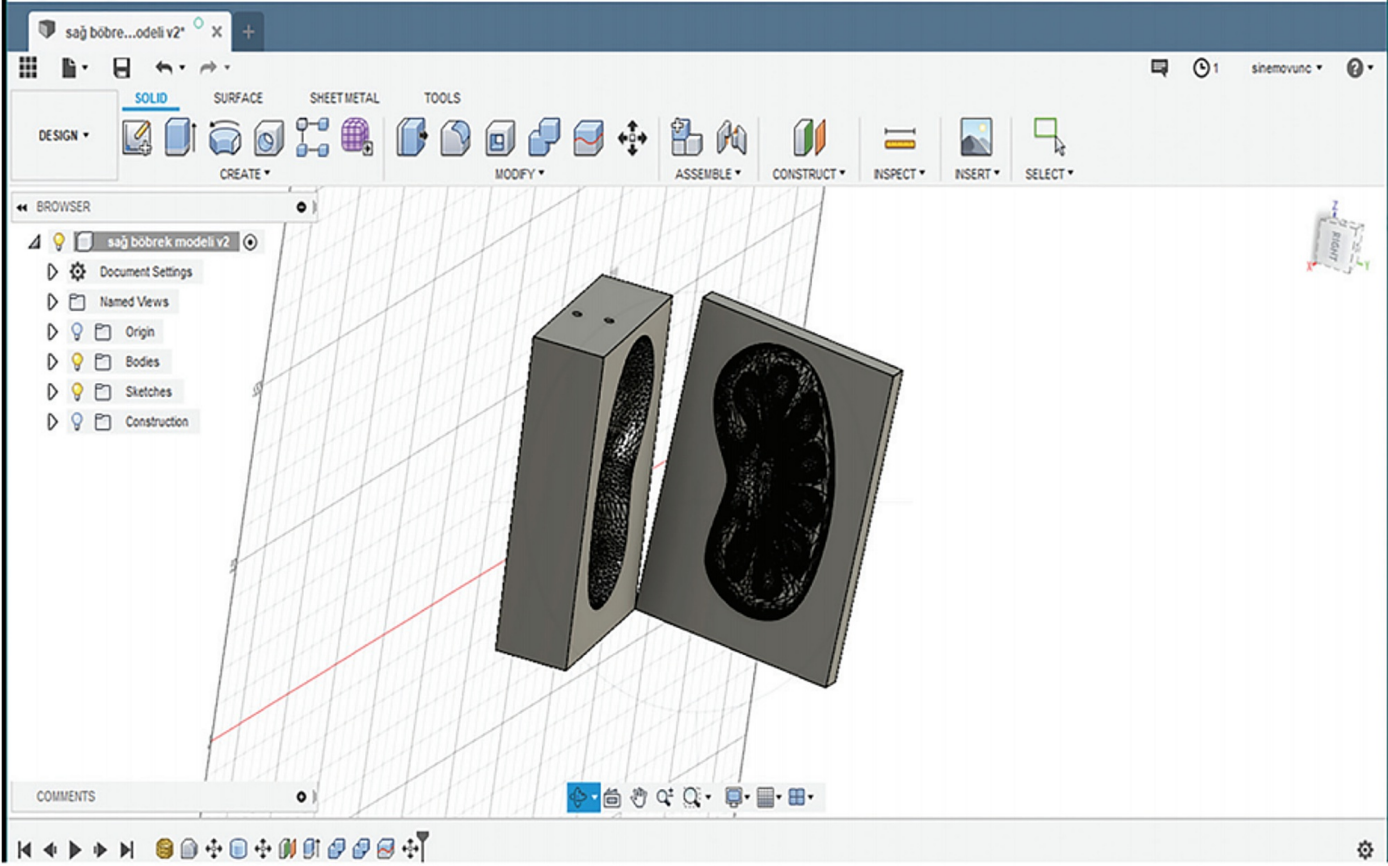
Designing the external mold The mold of the kidney model was prepared using the inverse of its geometry.

**Figure 7:**
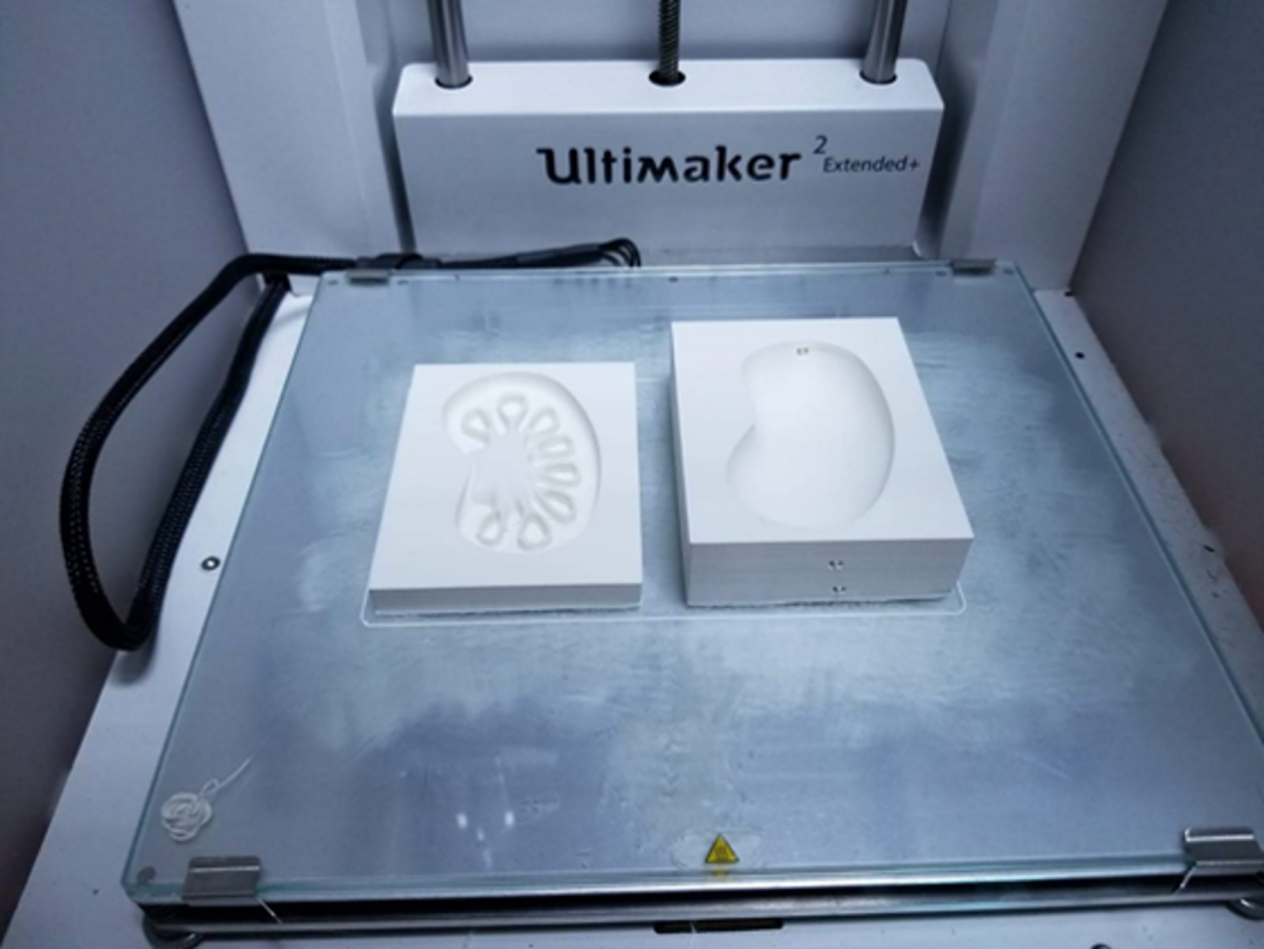
Fabrication of the external mold The mold of the kidney model was printed.

*(a) Design and Fabrication of the External Mold:* The virtual model of a kidney can be produced through different methods involving creating on CAD software and generating on 3D calculation software by using data acquired through digital imaging and communications in medicine (DICOM) from computed tomographic angiography (CTA) images. We received the virtual model of the kidney using these methods and imported it as an STL file into the CAD software (Fusion 360, Autodesk, San Rafael, USA) (Figure 5). The Create Base feature was selected to enter the direct editing mode. In this mode, due to the importing as an STL file into the software, the virtual model of the kidney was in a mesh form. However, the model allows users to create objects in another form named solid body. As a result, it is impossible to use the mesh form of the virtual model as reference geometry for the external mold. The mesh form of the virtual model was converted to the solid body using the Mesh to the B-Rep conversion tool. Then, the sketches, 2D geometries used as a base to construct 3D geometries, were created and used as paths to add three solid pipes (an inlet runner duct and two outlet vents) that facilitate the unidirectional filling of the kidney. A box was created according to the earlier designed parts of the mold to be the target body. The inverse geometry of the kidney and additional technical features were created by combining them with the target body using cut operation. In other words, firstly, the parts that would be hollow in the completed mold were created; then, they were excluded from the box that would be the body of the mold. Then the mold was split into two pieces using a horizontal plate to facilitate the removal of the kidney model. The completed external mold was exported and converted into an STL file (Figure 6). It was then 3D printed on the Ultimaker 2 Extended+ (Figure 7).

*(b) Preparation of the PVA Solution:* To prepare 20% w/v aqueous solution of PVA, 13.2 g of PVA powder was added to 47 mL of deionized water. The solution was heated and stirred at 100°C for approximately one hour until it became clear. The solution was left to cool to room temperature for about one hour. Finally, 19.6 mL of dimethyl sulfoxide (DMSO) was added to the solution. Throughout the preparation process, the solution was covered with stretch film to prevent drying.

*(c) Injection of the PVA Solution: *The constructed mold was sealed with silicone to prevent leaking during freeze/thaw cycles. The PVA solution was injected into the inlet runner duct of the mold using a 1 mL syringe until the overflow at the outlet vents was seen. Overall, 15 mL of PVA solution was used per model.

*d) The Freezing and Thawing Process:* Multiple freezing and thawing cycles were performed to convert the PVA solution into a gel. This allowed the material to form a porous network of hydrogen bonds and simulate characteristics of the parenchyma of the kidney.

The mechanical properties of the material were improved by altering the concentration of the solution and the number of freeze-thaw cycles based on feedback received from surgical experiences. Seven freeze-thaw cycles were subsequently administered to the entire external mold. Each cycle consists of two steps: (1) placing the mold in a −20°C freezer for 24 hours, and (2) thawing the mold at 4°C for 24 hours

**Figure 8:**
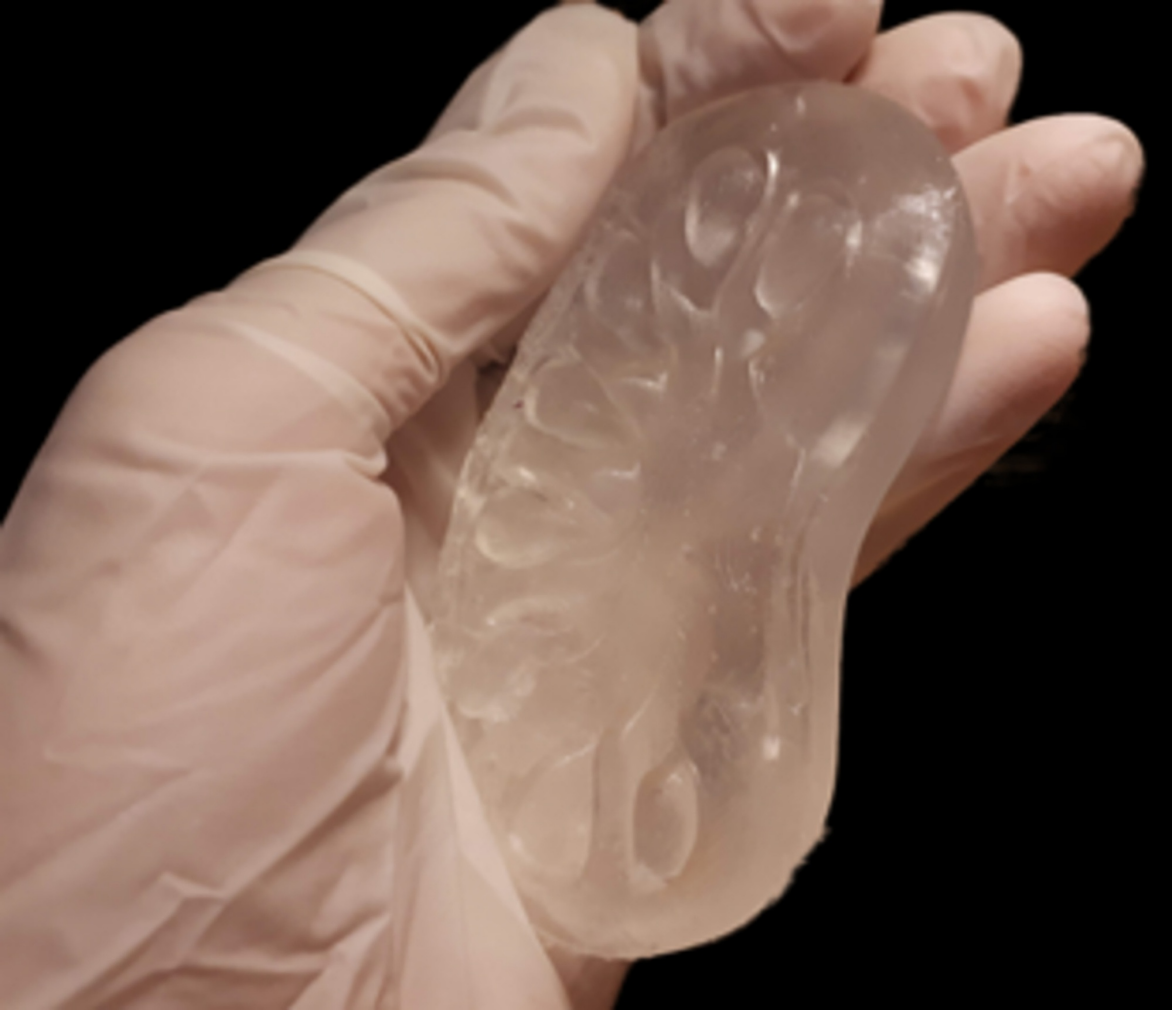
Kidney model based on 3D printing and hydrogel The completed kidney model was separated from the mold.

The completed model was then separated from the mold (Figure 8).

3. Creation of the hybrid pediatric surgery book based on AR

**Figure 9:**
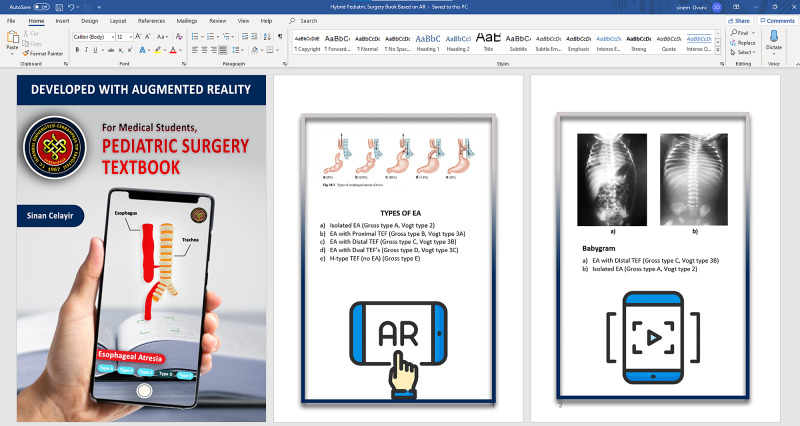
Compilation of the pediatric surgery book The pediatric surgery book was created for subjects including gastrointestinal atresias, ileus, malrotation, abdominal anomalies, and trauma.

*(a) Compilation of the Pediatric Surgery Book:* A pediatric surgery book was created from lecture notes prepared using reference textbooks for subjects involving gastrointestinal atresias, ileus, malrotation, abdominal anomalies, and trauma (Figure 9). Then parts that had been found appropriate for implementing AR were enhanced with visual instructions to make the book easier to use.

**Figure 10:**
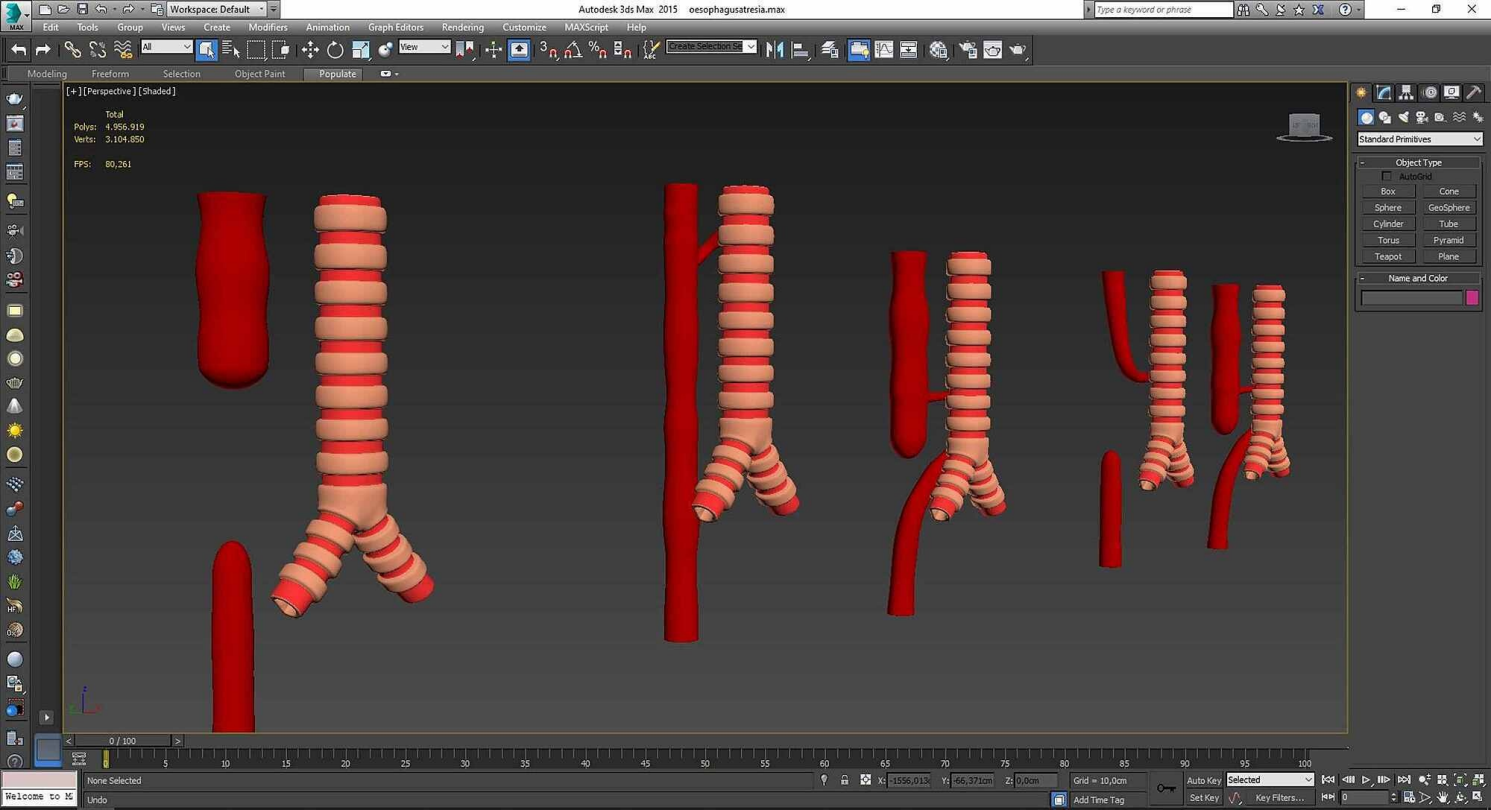
Creation of 3D models of related disorders using CAD programs Three-dimensional models of diseases regarding subjects constituted the pediatric surgery book were acquired. CAD: computer-aided design

*(b) Creation of 3D Models of Related Disorders Using Cad Programs: *3D models of diseases related to subjects in the pediatric surgery book created were acquired using Autodesk 3ds Max; then, they were revised in response to the feedback of pediatric surgeons (Figure 10).

*(c) Transformation of Radiologic Records Into Video Format: *Selected findings that would hold importance in the prognosis of diseases mentioned in the pediatric surgery book were highlighted on radiological views and recorded in video format.

*(d) AR Application Development: *Prepared 3D models and videos of radiological views were imported to a developer program named Unity (Unity Technologies, Copenhagen, Denmark). The platform of the AR application was acquired into Unity using the Vuforia tool. Required arrangements were made in the settings window of Unity.

There are two fundamental elements of AR application development. The first of them is the specificity of the activation of application in response to the detection of the desired surface or pattern. The second is the content, such as animation, 3D model, or video, that will be seen on the surface or pattern. At the first step of the application development, selected pages of the pediatric surgery book were used as an activating surface. In the next step, prepared 3D models and videos of radiological views were matched with activating surfaces in Unity. C# codes, which enabled users to move, magnify, or turn 3D models by finger movements, were written, and these codes, called scripts, were added to 3D models and videos to make our mobile application interactive.

**Figure 11:**
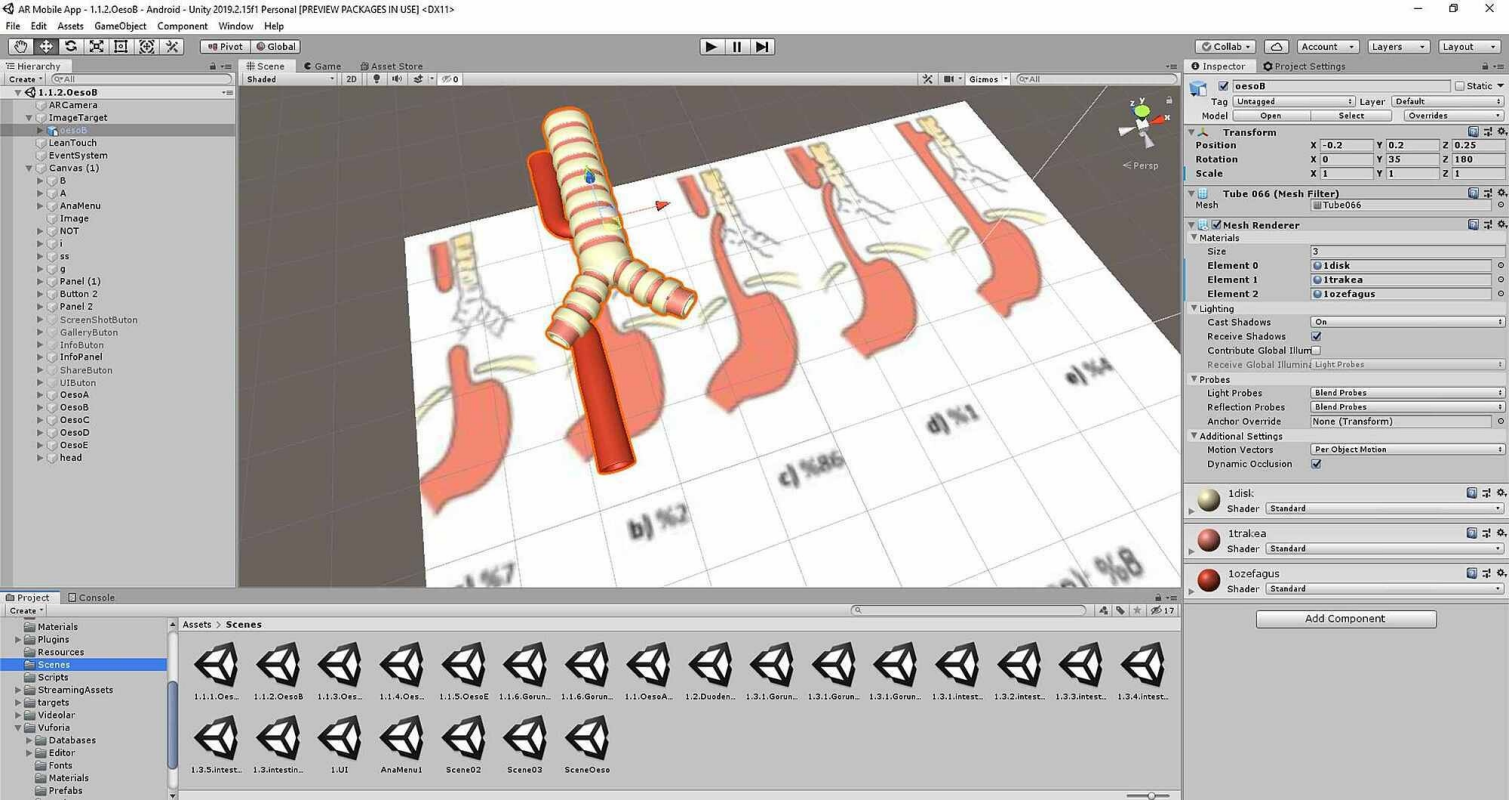
AR application development Accompanying Augmented Reality application was prepared. AR: Augmented Reality

Designs of the mobile application interface involving button designs and backgrounds were acquired using Adobe Photoshop CC (Adobe, Mountain View, California, USA) and implemented in the mobile app by importing into Unity. All of the prepared components were then combined (Figure 11).

Before launching the product in the market, we employed multiple methods to validate the functionality and identify bugs in the app. Following this, the final version of the app was tested on several devices with different resolutions. Then, we applied to launch our app in the market. It was offered as an open access app to students for testing.

4. Construction of the Mirror World Simulation

**Figure 12:**
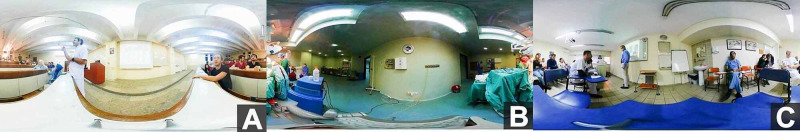
360-degree video recording 360-degree videos of three selected places involving classroom (A), operation room (B), and meeting room (C) were recorded.

*(a) 360-degree video recording:* 360-degree videos of three selected places, including classroom, operation room, and meeting room, were recorded (Figure 12 A, B, C). Then, video file sizes and color/light settings were adjusted using the Gear 360 video (Samsung, Seoul, Korea).

**Figure 13:**
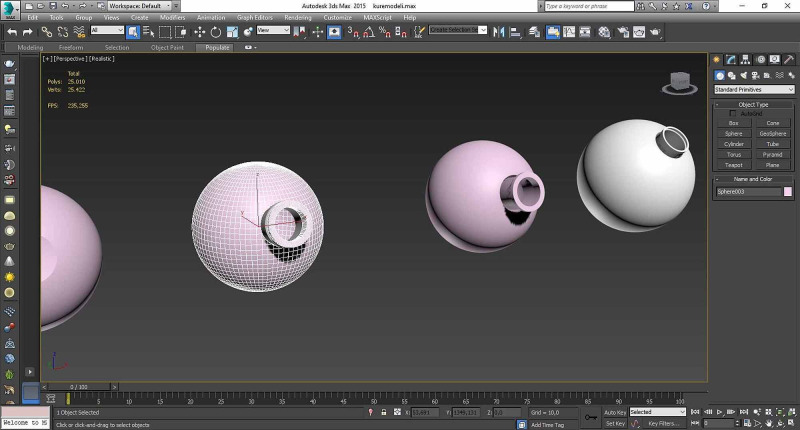
Construction of stages in the digital world Double-layer globes were acquired to use as stages for 360-degree videos.

*(b) Construction of Stages in the Digital World: *Double layer globes used as stages for 360-degree videos were acquired using Autodesk 3DsMax and positioned in a virtual environment (Figure 13).

**Figure 14:**
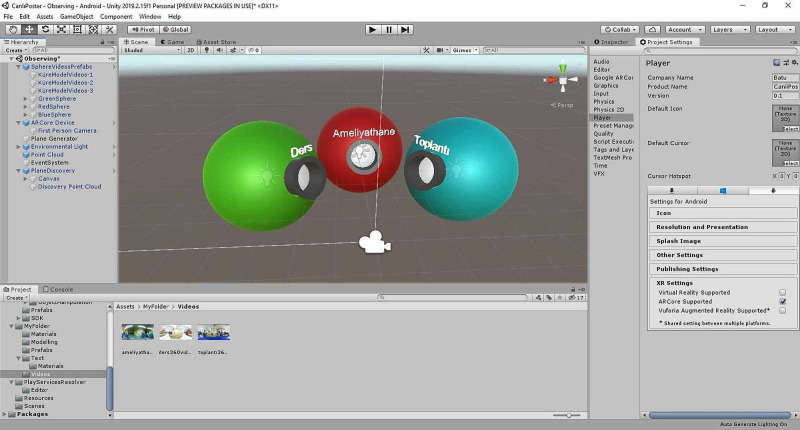
Construction of the Augmented Reality application

*(c) Combining 360-degree Videos With Globes: *360-degree videos were imported into Unity to combine with globes and compressed a second time. Then 360-degree videos were implemented into the inner layer of globes using the video tool of Unity, and virtual lights were added into each of the globes. Autoplay and Loop settings were selected (Figure 14).

*(d) Developing the AR Mobile App:* The Google ARCore platform was settled. AR Camera and surface detection scripts were added. 360-degree videos combined with globes were added as child files. Then, player settings that were required to develop the AR app in Unity were adjusted. The AR app was then finalized and built for Android devices and exported as .apk file.

## Discussion

3D-printed models of congenital anomalies

**Figure 15:**
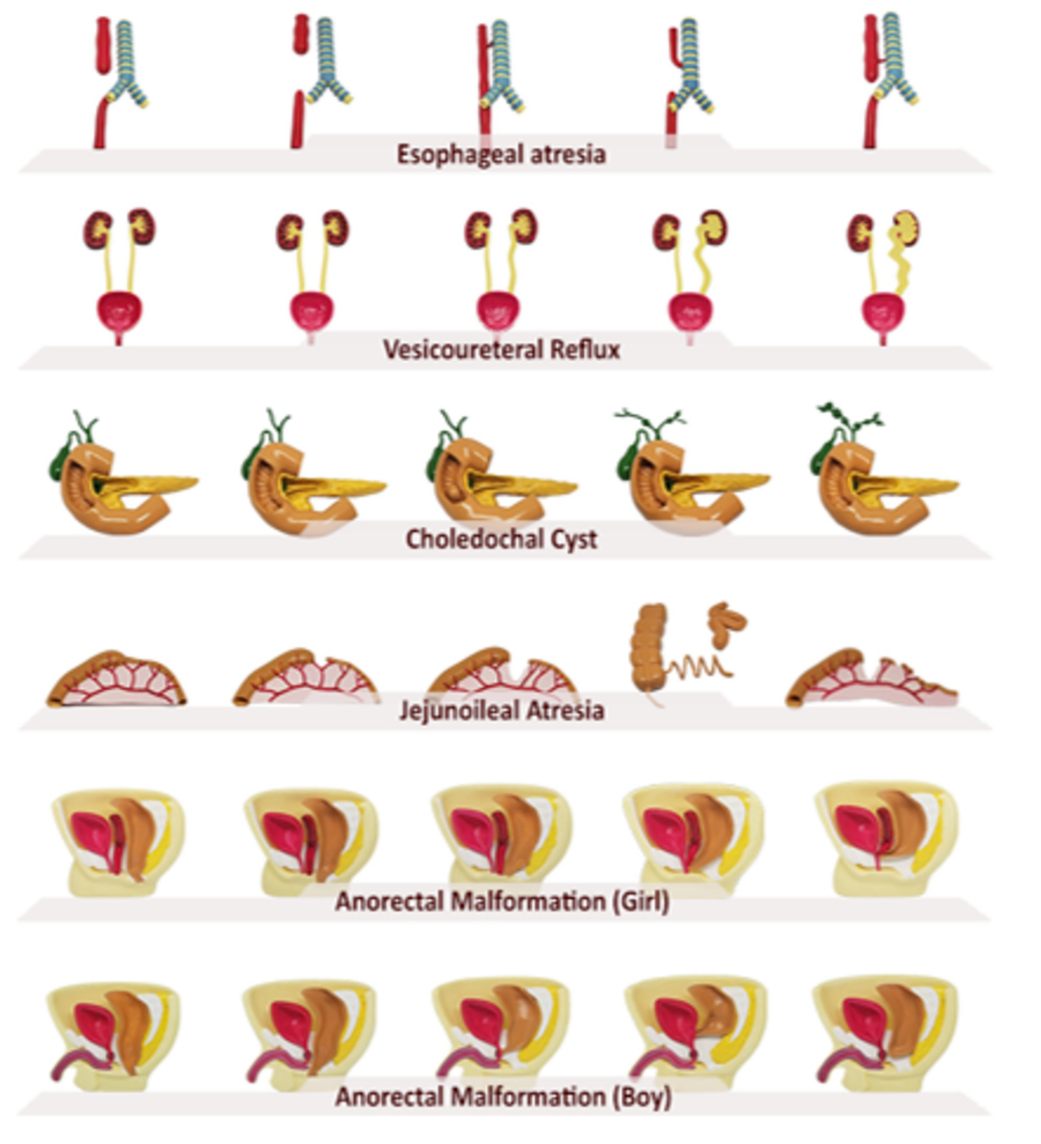
3D printed models of congenital anomalies

3D printing was the first immersive technology we used to construct educational materials. It has robust indications in health care involving dentistry, anatomical models, medical devices, tissue engineering scaffolds, tissue models, and drug formulation [10]. Particularly in surgical specialties, 3D printing has wide-range applications, including preoperative planning, patient education, patient-specific prostheses, orthoses and grafts, surgical devices, training, education, and case presentation [10-12]. In the present descriptive technical report, we utilized 3D printing to acquire models of five different pathologies in pediatric surgery, including anorectal malformation, esophageal atresia, vesicoureteral reflux, choledochal cyst, and jejunoileal atresia [7] (Figure 15).

3D printing enabled us to demonstrate complex anatomical relationships, implement active learning strategies in lectures, and construct case-specific and curriculum-specific models. The constructed models were used in pediatric surgery clerkship (Video 1).

**Video 1:** Surgical education materials were used in clerkships

Simulation models based on 3D printing and hydrogel

In addition to 3D printed models, we harnessed 3D printing to construct a flexible model of the kidney [9]. Contrary to the earlier models, for the kidney model, we used 3D printed molds acquired using inverse geometry and filled with PVA-based hydrogel as a tissue-mimicking material. Different materials are used in literature as tissue-mimicking material for training surgical procedures and preoperative planning [13-15]. Our preferred tissue-mimicking material and workflow led to flexible and low-cost models that enabled students to train in the fundamental surgical skills, such as suturing. However, further research is required to validate the characteristics of the tissue-mimicking material and the effectiveness of the kidney model. 

Hybrid pediatric surgery book based on AR

**Figure 16:**
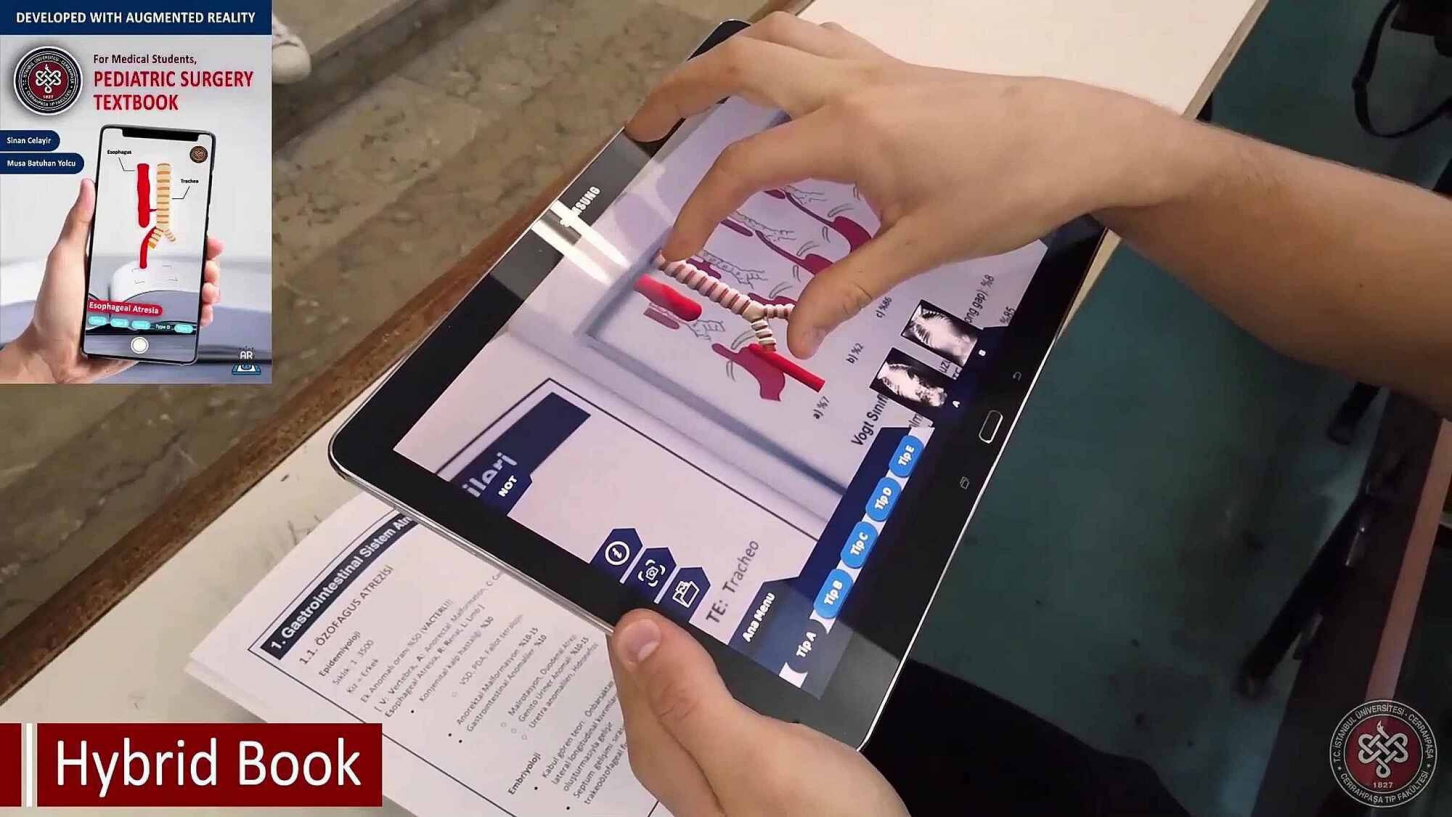
Hybrid pediatric surgery book based on AR

Even 3D printed models and hydrogel-based models had a third dimension: they weren't appropriate for "flipped classroom" or "self-paced learning" concepts and were limited in number. We constructed the hybrid pediatric surgery book based on AR to overcome these limitations. AR would be defined as a system that blends real and virtual worlds and enables users to perceive digital virtual visual content through real-life and real-time interaction. Compared to Virtual Reality, AR does not cut the connections of users with reality and surrounds them with a virtual world; instead, AR adds a layer onto real life. Another difference between AR and VR is device requirement. AR can be used with highly accessible devices, even with personal smartphones, whereas, VR requires relatively higher cost special devices, which was why we preferred to use AR to enhance the content of the books. The hybrid pediatric surgery book based on AR enabled students to examine spatial relationships and complicated pathologies and interact with them by walking around, zooming in/out, moving, and turning them. In addition to models, anonymized radiological views were added to the related content, which allowed students to easily access the radiologic views, look at their highlighted or raw versions, and examine them in detail by zooming in (Figure 16). More importantly, all content was usable through personal devices such as smartphones or tablets. Students were able to access models and radiological views when they wanted and where they wanted (Video 2). Despite the potential of AR to accompany the course curriculum, according to our knowledge, there are still very few examples in literature [8, 16-19]. However, we believe that enhancing the curriculum with AR would facilitate the learning of students and would be applicable in different surgical and non-surgical specialties with minor changes.

**Video 2:** A hybrid pediatric surgery book based on Augmented Reality was used in lectures

4. Mirror World Simulation

**Figure 17:**
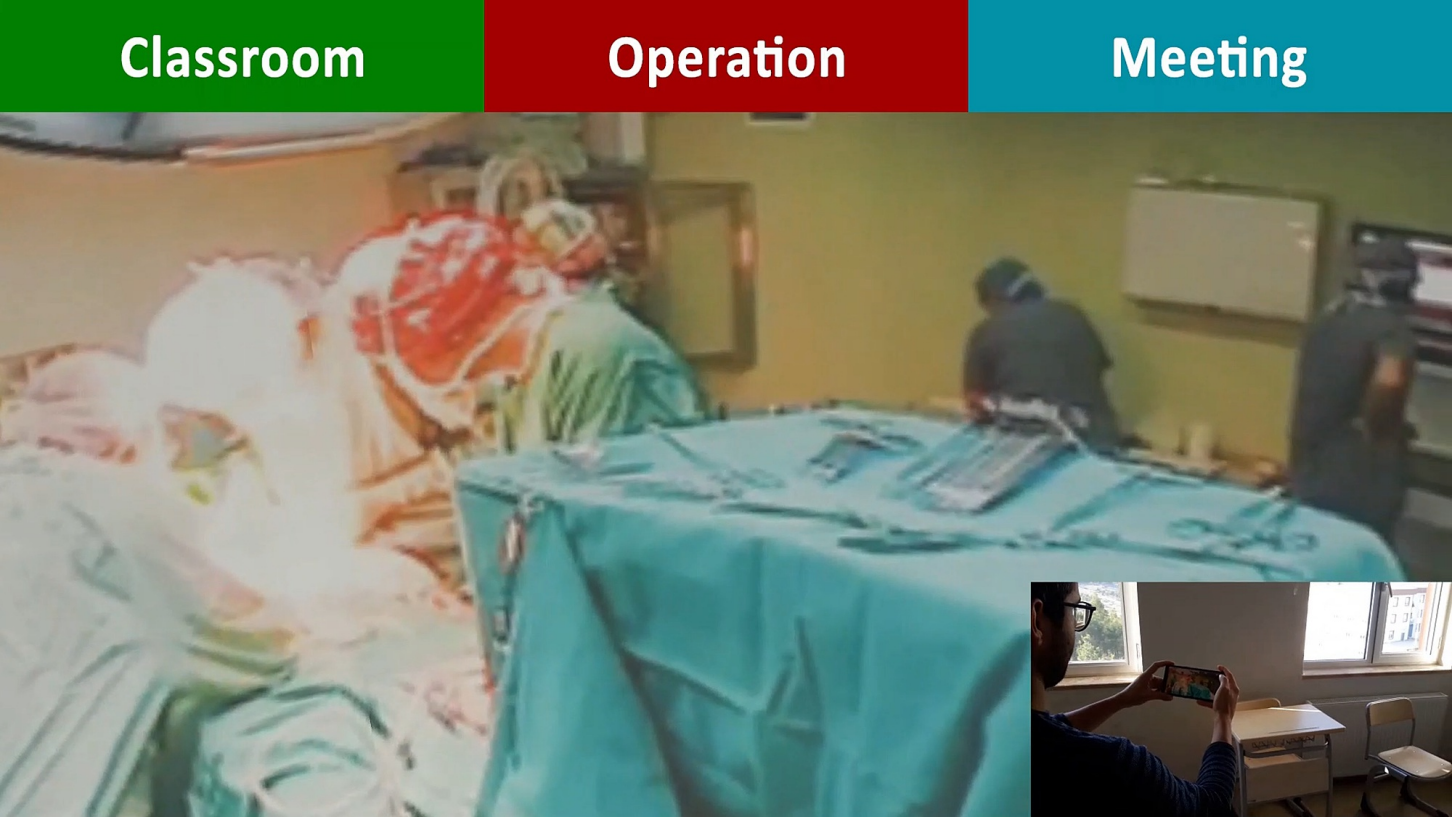
Mirror World Simulation Mirror World Simulation enabled students to experience different surgical environments.

Different internal and external factors involving restricted duty hours and a decreased indication of some surgical procedures led to a decrease in students' exposure to different surgical environments. The COVID-19 pandemic exacerbated that problem by limiting the number of surgeons in-house, case volumes, and in-person learning opportunities [16]. Mirror World is defined as a constructed copy of the real world in digital form [20]. We embedded 360-degree video recordings in Mirror World Simulation to acquire portals to different surgical settings including an operation room (OR), classroom, and a meeting room (Figure 17, Video 3). Using the operation room portal, students could experience the surgical procedure as if they were present in OR and review the surgical procedure from different angles. The classroom portal would increase student engagement in lectures, and the meeting room would help incorporate crowded student groups in meetings. We believe that Mirror World Simulation may be an effective solution for the new normal and may help the trainees on different levels, from undergrads to residents, to experience different surgical settings from various angles without time or place restriction.

**Video 3:** Mirror World Simulation enabled students to experience different surgical environments

## Conclusions

In our university, we constructed pediatric surgical education materials based on immersive technologies by the collaborative efforts of pediatric surgeons, lecturers, and medical students. We believe using immersive technologies in medical education may help lecturers and students in the new era with transforming pedagogy. In this paper, educational materials based on immersive technologies were described, and a detailed workflow was shared. We hope our workflow may serve as a valuable resource for other students and lecturers to develop their educational materials.

## References

[1] (2000). Looking back on the millennium in medicine. N Engl J Med.

[2] Hajar R (2011). Medical illustration: art in medical education. Heart Views.

[3] Guze PA (2015). Using technology to meet the challenges of medical education. Trans Am Clin Climatol Assoc.

[4] Eskander M, Neuwirth MG, Kuy S (2016). Technology for teaching: new tools for 21st century surgeons. Bull Am Coll Surg.

[5] Densen P (2011). Challenges and opportunities facing medical education. Trans Am Clin Climatol Assoc.

[6] Prensky M (2001). Digital natives, digital immigrants part 1. On the Horizon.

[7] Emre S, Yolcu MB, Celayir S (2018). Use of three dimensional printed models in the training of medical students in pediatric surgery: first impressions. J Turk Assoc Pediatr Surg Soc Pediatr Urol Turk.

[8] Yolcu MB, Emre S, Celayir S (2018). Use of augmented reality in medicine and pediatric surgery. J Turk Assoc Pediatr Surg Soc Pediatr Urol Turk.

[9] Ovunc SS, Yolcu MB, Emre S, Mammadov E, Celayir S (2019). Constructing low-cost simulation models in pediatric surgery and pediatric urology using 3 d printing and hydrogel: preliminary study. J Turk Assoc Pediatr Surg Soc Pediatr Urol Turk.

[10] Liaw CY, Guvendiren M (2017). Current and emerging applications of 3D printing in medicine. Biofabrication.

[11] Ganguli A, Pagan-Diaz GJ, Grant L (2018). 3D printing for preoperative planning and surgical training: a review. Biomed Microdevices.

[12] Transl A, Hoang D, Perrault D (2016). Surgical applications of three-dimensional printing: a review of the current literature & how to get started. Ann Transl Med.

[13] Shiraishi I, Yamagishi M, Hamaoka K, Fukuzawa M, Yagihara T (2010). Simulative operation on congenital heart disease using rubber-like urethane stereolithographic biomodels based on 3D datasets of multislice computed tomography. Eur J Cardio-thorac Surg.

[14] Ghazi AE, Teplitz BA (2020). Role of 3D printing in surgical education for robotic urology procedures. Transl Androl Urol.

[15] Cheung CL, Looi T, Lendvay TS, Drake JM, Farhat WA (2014). Use of 3-dimensional printing technology and silicone modeling in surgical simulation: development and face validation in pediatric laparoscopic pyeloplasty. J Surg Educ.

[16] Keller DS, Grossman RC, Winter DC (2020). Choosing the new normal for surgical education using alternative platforms. Surgery (Oxf).

[17] Tang KS, Cheng DL, Mi E, Greenberg PB (2020). Augmented reality in medical education: a systematic review. Can Med Educ J.

[18] Billinghurst M, Kato H, Poupyrev I (2001). The MagicBook: a transitional AR interface. Comput Graph.

[19] Leydon GB, Schwartz ML (2020). The use of mobile devices to enhance engagement and integration with curricular content. Yale J Biol Med.

[20] Gelernter D (1991). Mirror Worlds: Or the Day Software Puts the Universe in a Shoebox ... How It Will Happen and What It Will
Mean. https://books.google.com.tr/books/about/Mirror_Worlds.html?id=jh2U379fq18C&redir_esc=y.

